# Supplementary material for: Transformation of Combustion Nanocatalysts inside Solid Rocket Motor under Various Pressures
Source: Nanomaterials (Basel). 2019 Mar 6;9(3):381. doi: 10.3390/nano9030381 (PMC6473950; doi:10.3390/nano9030381)
Supplement: Supplementary file 1 [file nanomaterials-09-00381-s001.pdf]

Supplementary Materials

Transformation of Combustion Nanocatalysts inside Solid Rocket Motor under Various Pressures

Jun-Qiang Li [1,†], Linlin Liu [2,†], Xiaolong Fu [1], Deyun Tang [2], Yin Wang [2], Songqi Hu [2] and Qi-Long Yan [2,*]

1 Xi'an Modern Chemistry Research Institute, Xi'an 710065, China; llijq@sohu.com (J.-Q.L.); fuxiaolong204@163.com (X.F.)
2 Science and Technology on Combustion, Internal Flow and Thermo-structure Laboratory, Northwestern Polytechnical University, Xi'an 710072, China; lll@nwpu.edu.cn (L.L.); tangdy@mail.nwpu.edu.cn (D.T.); wongyin@mail.nwpu.edu.cn (Y.W.); pinecore@nwpu.edu.cn (S.H.)
† These authors contribute equally to this work.
* Correspondence: qilongyan@nwpu.edu.cn

Characterizations

The surface morphology and microstructure of the condensed combustion products were examined by means of scanning electron microscopy (SEM). The SEM analysis was operated on a Quanta FEG 250, with an accelerating voltage 15 or 20 kV. Meanwhile, the products have to be coated by gold to get better electron conductivity. The field emission SEM is coupled with an accessory Energy Dispersive Spectrometer (EDS), through which we can get the element distribution information of the condensed products. More detailed compositions and structure information can be obtained by Transmission electron microscopy (TEM). The TEM analysis was operated on a Tecnai G2 F20, by which the target samples were dispersed in ethanol, so that they can achieve a maximum magnification of 1 million times at an accelerating voltage of 200 kV. A laser particle size analyzer (Kurt LS13320) was used to measure the particle size distribution of the involved combustion products. The compositions of the samples and the crystal phases of the elements were characterized by powder X-ray diffraction (XRD, Panaco Sharp Xpert Pro MPD; Bruker C2 Discover with GADDS, operating at 40 kV and 40mA with unfiltered Cu Ka radiation, E1/48049 eV, k1/41.5406 Å).

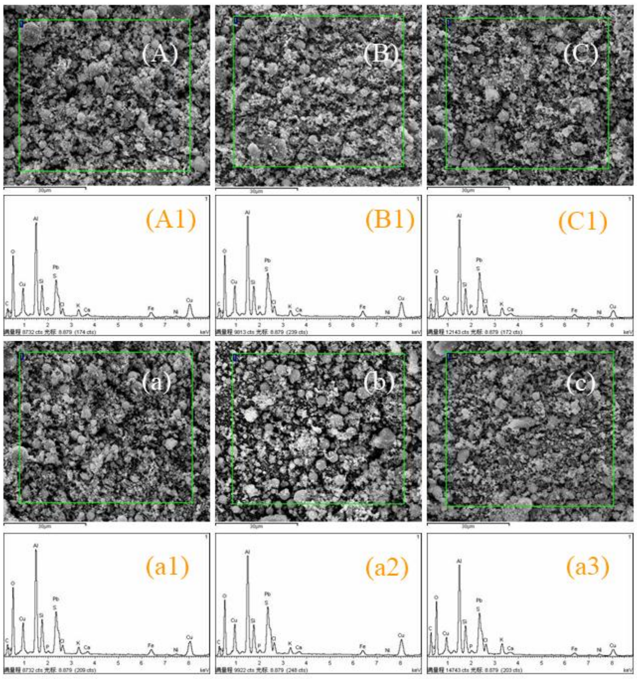

Figure S1. The EDS spectra of CCPs from JZ propellant grains.

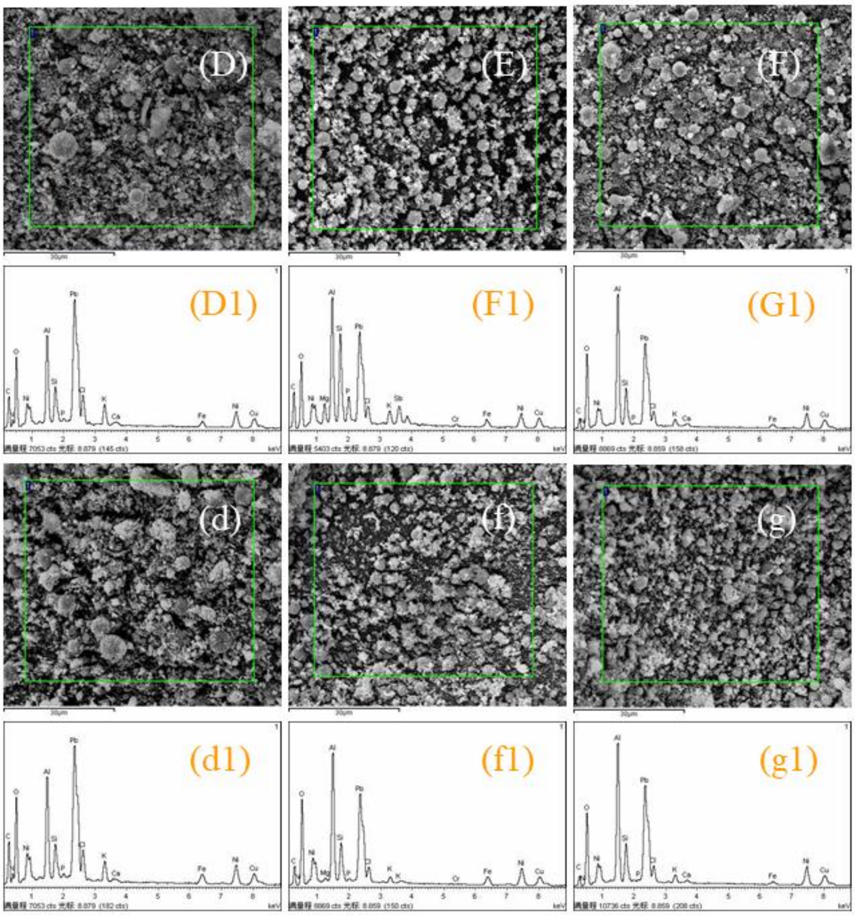

Figure S2. The EDS spectra of CCPs from LZ propellant grains.

Table S1. Element distribution results of JZ and LZ propellant grains combustion products.

		**Test 1**				**Test 2**			
**Samples**	**Element**	**Element concentration**	**Weight Percentage**	**Weight Percentage (Sigma)**	**atom Percentage**	**Element concentration**	**Weight Percentage**	**Weight Percentage (Sigma)**	**atom Percentage**
JZ-15	C K	6.27	0.3789	1.10	29.56	6.82	0.3808	1.06	30.98
	N K	0.37	0.1636	0.89	3.42	0.36	0.1615	0.88	3.33
	O K	23.40	0.7110	0.61	44.14	23.48	0.7040	0.59	43.34
	Al K	9.04	0.7491	0.23	9.60	9.29	0.7544	0.22	9.49
	Si K	3.21	0.7492	0.11	3.27	3.21	0.7532	0.10	3.15
	P K	0.42	1.1293	0.05	0.26	0.38	1.1353	0.05	0.23
	S K	1.37	0.8614	0.10	1.06	1.52	0.8644	0.10	1.14
	Cl K	0.79	0.6965	0.07	0.69	0.83	0.6958	0.06	0.70
	K K	1.33	0.9784	0.06	0.75	1.13	0.9768	0.06	0.61
	Ca K	0.42	0.9487	0.05	0.24	0.42	0.9481	0.05	0.23
	Fe K	2.29	0.8912	0.11	0.99	2.38	0.8890	0.11	1.00
	Ni K	0.71	0.8807	0.12	0.30	0.61	0.8797	0.11	0.25
	Cu K	10.99	0.8403	0.32	4.42	10.84	0.8393	0.30	4.23
	Pb M	9.91	0.7794	0.37	1.32	10.38	0.7819	0.36	1.33
JZ-20	C K	5.58	16.18	1.03	30.23	6.45	16.67	1.01	31.94
	N K	0.28	1.86	0.82	2.98	0.24	1.42	0.82	2.33
	O K	20.19	30.57	0.55	42.89	20.73	28.35	0.52	40.79
	Al K	8.41	12.08	0.22	10.05	9.58	12.09	0.22	10.31
	Si K	2.66	3.82	0.10	3.05	2.96	3.73	0.09	3.06
	P K	0.47	0.45	0.05	0.33	0.56	0.46	0.05	0.34
	S K	1.37	1.70	0.10	1.19	1.69	1.85	0.10	1.32

	Cl K	0.95	1.47	0.07	0.93	1.23	1.70	0.07	1.11
	K K	0.92	1.01	0.05	0.58	1.14	1.11	0.06	0.66
	Ca K	0.34	0.38	0.05	0.21	0.38	0.38	0.05	0.22
	Fe K	2.11	2.53	0.11	1.02	1.91	2.02	0.10	0.83
	Ni K	0.45	0.54	0.10	0.21	0.54	0.57	0.10	0.22
	Cu K	10.82	13.74	0.30	4.85	12.80	14.23	0.31	5.15
	Pb M	9.94	13.66	0.36	1.48	12.77	15.42	0.38	1.71
JZ-25	C K	11.67	24.75	0.93	42.36	21.02	30.07	0.73	46.50
	N K	0.38	2.36	0.95	3.47	0.58	3.02	0.90	4.00
	O K	18.85	26.94	0.51	34.61	26.47	30.34	0.48	35.22
	Al K	11.18	11.71	0.21	8.92	11.31	9.11	0.14	6.27
	Si K	3.32	3.56	0.09	2.61	3.76	3.03	0.07	2.01
	P K	0.83	0.59	0.05	0.39	0.63	0.34	0.03	0.20
	S K	1.57	1.51	0.09	0.97	2.00	1.47	0.07	0.85
	Cl K	1.45	1.80	0.07	1.04	1.38	1.28	0.05	0.67
	K K	1.69	1.51	0.06	0.79	2.53	1.71	0.05	0.81
	Ca K	0.49	0.45	0.05	0.23	0.44	0.31	0.03	0.15
	Fe K	1.32	1.30	0.08	0.48	1.34	1.04	0.06	0.35
	Ni K	0.81	0.80	0.10	0.28	1.24	0.96	0.08	0.30
	Cu K	6.93	7.13	0.21	2.31	6.75	5.49	0.15	1.61
	Pb M	14.58	15.58	0.37	1.55	14.51	11.83	0.27	1.06
LZ-7	C K	12.52	21.23	0.88	39.09	14.02	23.35	0.82	41.17
	N K	1.62	8.81	0.96	13.91	1.62	9.05	0.93	13.68
	O K	14.87	22.75	0.48	31.45	15.06	23.48	0.46	31.09
	Al K	4.79	5.23	0.11	4.28	4.61	5.13	0.10	4.03
	Si K	2.13	2.15	0.07	1.69	1.69	1.75	0.06	1.32
	P K	0.38	0.25	0.04	0.18	0.38	0.26	0.04	0.18
	Cl K	1.77	2.21	0.08	1.38	1.55	1.94	0.07	1.16
	K K	2.16	1.94	0.07	1.09	1.80	1.62	0.06	0.88
	Ca K	0.35	0.32	0.05	0.18	0.18	0.16	0.04	0.09
	Fe K	1.48	1.42	0.09	0.56	2.02	1.98	0.09	0.75
	Ni K	6.09	5.81	0.18	2.19	6.02	5.89	0.16	2.13
	Cu K	4.20	4.19	0.18	1.46	3.99	4.09	0.16	1.36
	Pb M	23.28	23.68	0.46	2.53	20.57	21.30	0.40	2.18
LZ-18	C K	10.03	26.05	0.79	49.22	8.67	18.11	0.66	35.44
	N K	3.03	4.62	0.18	0.86	0.24	1.29	0.48	2.16
	O K	11.30	19.92	0.41	28.25	21.42	27.88	0.40	40.95
	Mg K	0.87	1.45	0.07	1.35	0.19	0.27	0.05	0.26
	Al K	5.24	7.58	0.14	6.37	8.25	9.90	0.14	8.62
	Si K	3.97	5.47	0.11	4.42	2.67	3.12	0.08	2.61
	P K	2.01	1.88	0.08	1.38	0.35	0.26	0.05	0.20
	Cl K	0.81	1.33	0.08	0.85	1.02	1.38	0.07	0.92
	K K	1.15	1.33	0.07	0.77	0.72	0.69	0.05	0.42
	Cr K	0.29	0.38	0.08	0.17	0.07	0.07	0.06	0.03
	Fe K	1.37	1.73	0.11	0.70	2.38	2.41	0.10	1.01
	Ni K	3.96	4.97	0.18	1.92	7.63	7.74	0.18	3.10
	Cu K	3.63	4.75	0.20	1.70	4.52	4.79	0.18	1.77
	Pb M	13.19	18.54	0.38	2.03	19.50	22.09	0.35	2.51
LZ-35	C K	4.92	12.39	1.05	26.48	4.93	11.38	0.90	25.67
	N K	0.24	1.36	0.81	2.49	0.22	1.11	0.69	2.16
	O K	19.54	27.80	0.52	44.61	19.74	25.65	0.43	43.43
	Al K	8.93	12.00	0.22	11.42	9.84	12.13	0.19	12.18
	Si K	2.74	3.64	0.10	3.33	2.98	3.62	0.09	3.49
	P K	0.17	0.15	0.05	0.12	0.17	0.14	0.05	0.12
	Cl K	1.04	1.61	0.08	1.17	1.09	1.56	0.08	1.19
	K K	0.91	0.98	0.06	0.65	1.09	1.09	0.06	0.75

Ca K	0.38	0.42	0.05	0.27	0.36	0.36	0.05	0.25
Fe K	1.15	1.29	0.10	0.59	0.95	0.96	0.09	0.47
Ni K	7.05	7.88	0.22	3.45	8.95	9.00	0.20	4.15
Cu K	5.08	5.94	0.22	2.40	5.90	6.19	0.20	2.64
Pb M	19.18	24.54	0.48	3.04	23.08	26.82	0.44	3.51

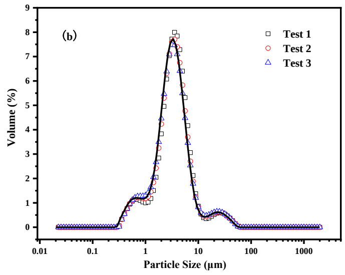

JZ-7

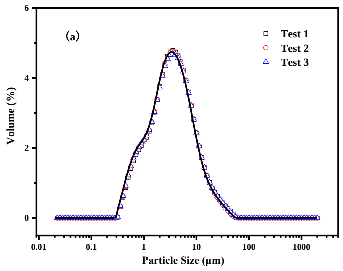

JZ-12

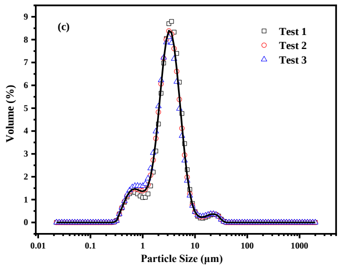

JZ-15

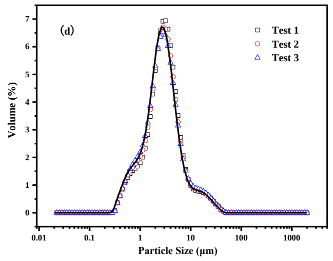

JZ-20

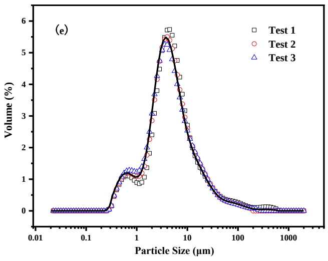

JZ-25

Figure S3. The particle size distribution curves for JZ propellant grains combustion under various pressures with repeated tests.

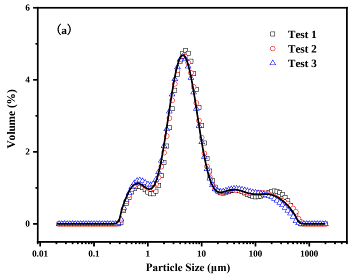

LZ-0

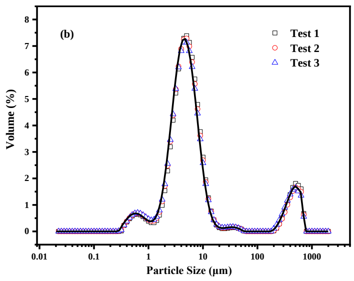

LZ-2

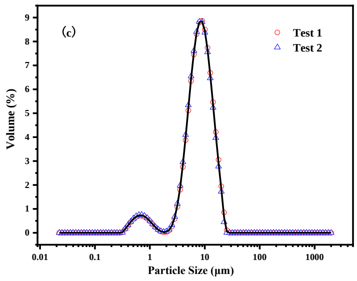

LZ-5

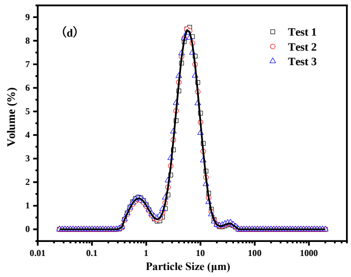

LZ-7

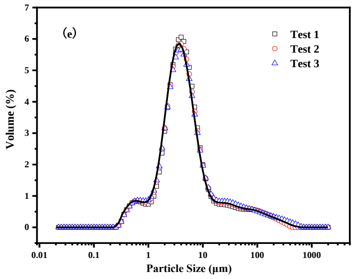

LZ-9

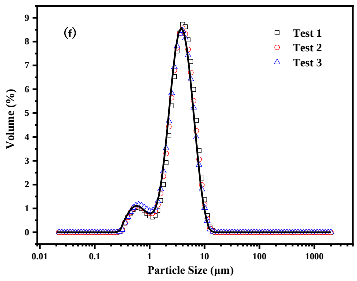

LZ-12

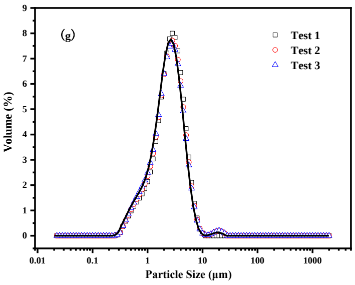

LZ-18

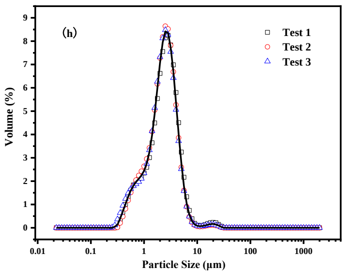

LZ-35

Figure S4. The particle size distribution curves for LZ propellant grains combustion under various pressures with repeated tests.

Table S2. A summary of the CCPs particle distributions for JZ propellant grains.

Samples		Obscuration	Residual	Concentration	Span	D[4,3]	Uniformity	SSA	D[3, 2] - Surface weighted mean	d(0.1)	d(0.5)	d(0.9)
JZ-7	Test1	7.63	1.43	0.0027	3.157	5.045	1.06	3.03	1.981	0.806	3.202	10.915
	Test2	7.6	1.204	0.0027	3.118	4.901	1.03	3.07	1.951	0.792	3.167	10.663
	Test3	7.58	1.317	0.0027	3.188	4.998	1.06	3.09	1.944	0.785	3.169	10.887
	mean	7.6	1.317	0.0027	3.154	4.981	1.05	3.06	1.959	0.794	3.179	10.819
JZ-12	Test1	8.88	1.261	0.0034	1.879	4.654	0.801	2.68	2.239	1.104	3.293	7.292
	Test2	8.9	1.043	0.0033	2.005	4.569	0.847	2.79	2.153	1.025	3.144	7.33
	Test3	8.91	1.127	0.0032	2.098	4.541	0.882	2.84	2.114	0.995	3.061	7.418
	mean	8.9	1.144	0.0033	1.992	4.588	0.842	2.77	2.167	1.035	3.167	7.343
JZ-15	Test1	8.53	1.845	0.003	1.61	3.596	0.571	2.93	2.045	0.95	3.03	5.827
	Test2	8.56	1.801	0.0029	1.693	3.466	0.61	3.1	1.933	0.87	2.851	5.697
	Test3	8.57	1.749	0.0028	1.746	3.386	0.633	3.22	1.866	0.831	2.745	5.623
	mean	8.55	1.798	0.0029	1.685	3.483	0.606	3.08	1.945	0.875	2.876	5.721
JZ-20	Test1	9.3	1.125	0.0031	2.385	4.004	0.87	3.15	1.906	0.865	2.742	7.403
	Test2	9.31	1.177	0.003	2.517	3.937	0.906	3.25	1.846	0.83	2.646	7.492
	Test3	9.31	1.073	0.003	2.646	3.937	0.941	3.31	1.814	0.813	2.592	7.671
	mean	9.31	1.125	0.003	2.512	3.959	0.905	3.24	1.855	0.834	2.661	7.519
JZ-25	Test1	8.37	0.608	0.0039	4.079	12.349	2.27	2.35	2.556	1.099	4.382	18.972
	Test2	8.48	0.634	0.0037	3.853	8.077	1.45	2.51	2.386	0.982	4.055	16.606
	Test3	8.53	0.627	0.0036	4.041	8.274	1.59	2.61	2.301	0.926	3.903	16.696
	mean	8.46	0.623	0.0037	3.967	9.567	1.78	2.49	2.41	0.99	4.116	17.317

Table S3. Particle distribution of LZ propellant grains combustion products.

Samples		Residual	Concentration	Span	D [4, 3] - Volume weighted mean	Uniformity	SSA	D [3, 2] - Surface weighted mean	d (0.1)	d (0.5)	d (0.9)
LZ-0	Test 1	0.570	0.0047	20.455	36.345	5.91	1.98	3.024	1.235	5.654	116.882
	Test 2	0.559	0.0044	19.948	35.469	6.22	2.14	2.804	1.075	5.274	106.282
	Test 3	0.585	0.0042	16.814	28.885	5.31	2.26	2.65	0.988	4.97	84.558
	mean	0.571	0.0044	18.859	33.566	5.83	2.13	2.818	1.084	5.298	101.003
LZ-2	Test 1	0.764	0.0058	63.939	56.990	11.00	1.77	3.390	2.092	4.886	314.492
	Test 2	0.923	0.0057	58.168	53.230	10.60	1.84	3.253	1.975	4.753	278.462
	Test 3	0.908	0.0057	60.503	54.282	10.90	1.86	3.22	1.921	4.723	287.694
	mean	0.865	0.0057	61.074	54.834	10.80	1.83	3.286	1.995	4.788	294.406
LZ-5	Test 1	1.694	0.0079	1.442	9.195	0.459	0.85	7.070	4.15	7.956	15.624
	Test 2	1.53	0.0064	1.382	8.291	0.435	1.38	4.361	3.568	7.7	14.213
	Test 3	1.618	0.0063	1.385	8.043	0.436	1.43	4.203	3.409	7.5	13.797
	mean	1.614	0.0069	1.392	8.510	0.445	1.22	4.929	3.761	7.714	14.498
LZ-7	Test 1	1.946	0.0043	1.698	5.224	0.514	2.33	2.573	0.947	4.72	8.962
	Test 2	2.040	0.0043	1.686	5.153	0.527	2.31	2.600	1.069	4.578	8.790
	Test 3	1.969	0.0041	1.732	5.000	0.546	2.42	2.477	0.96	4.387	8.557
	mean	1.985	0.0042	1.708	5.126	0.530	2.35	2.549	0.986	4.561	8.777
LZ-9	Test 1	0.452	0.0047	6.834	14.787	3.060	2.28	2.637	1.358	4.072	29.182

	Test 2	0.453	0.0046	7.076	14.091	2.92	2.30	2.604	1.295	4.037	29.859
	Test3	0.542	0.0047	8.194	17.396	3.710	2.29	2.617	1.276	4.073	34.649
	mean	0.482	0.0047	7.373	15.425	3.230	2.29	2.619	1.308	4.06	31.245
LZ-12	Test 1	0.650	0.0036	1.496	3.898	0.453	2.54	2.362	1.321	3.619	6.737
	Test 2	0.702	0.0035	1.545	3.739	0.465	2.66	2.259	1.168	3.461	6.517
	Test 3	0.871	0.0035	1.569	3.637	0.470	2.73	2.195	1.087	3.364	6.366
	mean	0.741	0.0035	1.541	3.758	0.464	2.64	2.270	1.18	3.481	6.544
LZ-18	Test 1	1.482	0.0028	1.586	2.643	0.485	3.45	1.740	0.873	2.402	4.683
	Test 2	1.618	0.0027	1.654	2.713	0.548	3.53	1.698	0.836	2.345	4.714
	Test 3	1.583	0.0027	1.675	2.673	0.559	3.61	1.663	0.812	2.298	4.661
	mean	1.561	0.0027	1.638	2.676	0.53	3.53	1.700	0.839	2.349	4.685
LZ-35	Test 1	1.619	0.0029	1.607	2.653	0.562	3.70	1.623	0.757	2.279	4.419
	Test 2	1.744	0.0028	1.504	2.425	0.503	3.74	1.605	0.785	2.168	4.045
	Test 3	1.844	0.0028	1.568	2.410	0.527	4.00	1.501	0.703	2.132	4.047
	mean	1.736	0.0028	1.562	2.496	0.533	3.81	1.574	0.75	2.191	4.171

Notes: a), the weighted average of the particle size to the surface area; b), the weighted average of the particle size to the volume. c), particles whose diameter is equal to or less than the value of D(0.1), and the sum of the volume fractions of which accounts for ten percent ; it is the same for d) and e); SSA, specific surface area.

Table S4. Chemical compositions of the CCPs of JZ propellants at various pressures.

	Chemicals (volume %)								
**Samples**	**Cu**	**Pb(OH)Cl**	**$Pb_2Cl_2(CO_3)$**	**Al_2O**	**$AlCu_3$**	**$AlCu_4$**	**Al_2O_3**	C	SiO_2
JZ-7	46	36	-	18	-	-	-	-	-
JZ-12	43	16	-	-	14	-	-	-	27
JZ-15	34	34	-	-	-	32	-	-	-
JZ-20	37		50	-	-	-	13	-	-
JZ-25			29	-	25	-	-	46	-

Table S5. Chemical compositions of the CCPs of LZ propellants at various pressures.

**Samples**	**LZ-0**	**LZ-2**	**LZ-5**	**LZ-7**	**LZ-9**	**LZ-12**	**LZ-18**	**LZ-35**
Cu	-	40	-	-	-	-	-	-
CuO	5	-	-	-	-	-	-	-
Cu_3N_1	-	-	18	26	-	-	-	-
$Cu_{1.8}S$	-	-	-	-	-	-	21	-
$C_{12}H_{27}N_3O_6 \bullet CuC_{12} \bullet 2H_2O$	-	-	-	-	12	-	-	-
$Cu(NO_3)_2 \bullet 3H_2O$	-	-	-	-	-	-	-	13
Pb(OH)Cl	34	27	31	-	47	-	-	51
Pb_2SO_5	-	-	-	27	-	-	-	-
$2Pb(CO_3) \bullet Pb(OH)_2$	-	-	-	-	-	32	-	-
$Pb_4O_3Cl_2 \bullet H_2O$	-	-	-	-	-	-	23	
Al_2O_3	37		29	23	-	-	-	-
$AlNi_3C_{0.5}$	-	35	-	-	-	-	-	-
$HAlO_{10}Si_4$	-	-	-	-	30	-	-	-
$AlCu_4$	-	-	-	-	-	20	-	-
AlN	-	-	-	-	-	-	27	-
$CH_{18}AlN_3O_{14}S_2$	-	-	-	-	-	-	-	11
Ni	30	-	-	-	-	-	29	-
$Pb_2Ni(NO_2)_6$	-	-	19	-	-	-	-	-
$(NH_4)_2Ni(SO_4)_2 \bullet 6H_2O$	-	-	-	24	-	-	-	-
$C_2H_2NiO_4 \bullet 2H_2O$	-	-	-	-	11	-	-	-
NiO	-	-	-	-	-	20	-	25
C	-	-	-	-	-	28	-	-
